# Children Living near a Sanitary Landfill Have Increased Breath Methane and *Methanobrevibacter smithii* in Their Intestinal Microbiota

**DOI:** 10.1155/2014/576249

**Published:** 2014-10-13

**Authors:** Humberto Bezerra de Araujo Filho, Mirian Silva Carmo-Rodrigues, Carolina Santos Mello, Lígia Cristina Fonseca Lahoz Melli, Soraia Tahan, Antonio Carlos Campos Pignatari, Mauro Batista de Morais

**Affiliations:** ^1^Division of Pediatric Gastroenterology, Universidade Federal de São Paulo, 598 Botucatu Street, Vila Clementino, 04023-062 São Paulo, SP, Brazil; ^2^Centro Universitário FIEO, 300 Franz Voegeli Avenida, Vila Yara, 06020-190 Osasco, SP, Brazil; ^3^Division of Medicine, Universidade Federal de São Paulo, 188 Leandro Dupret Street, Vila Clementino, 04025-010 São Paulo, SP, Brazil

## Abstract

This study evaluated the breath CH_4_ excretion and concentration of *M. smithii* in intestinal microbiota of schoolchildren from 2 slums. One hundred and eleven children from a slum near a sanitary landfill, 35 children of a slum located away from the sanitary landfill, and 32 children from a high socioeconomic level school were included in the study. Real-time PCR was performed to quantify the *M. smithii nifH* gene and it was present in the microbiota of all the participating children, with higher (*P* < 0.05) concentrations in those who lived in the slum near the landfill (3.16 × 10^7^ CFU/g of feces), comparing with the children from the slum away from the landfill (2.05 × 10^6^ CFU/g of feces) and those from the high socioeconomic level group (3.93 × 10^5^ CFU/g of feces). The prevalence of children who present breath methane was 53% in the slum near the landfill, 31% in the slum further away from the landfill and, 22% in the high socioeconomic level group. To live near a landfill is associated with higher concentrations of *M. smithii* in intestinal microbiota, comparing with those who live away from the landfill, regardless of their socioeconomics conditions.

## 1. Introduction

The human intestinal microbiota consists of a diverse group of microorganisms that play an important role in controlling the colonization of the gastrointestinal tract and the maturation and proliferation of intestinal cells as well as regulating the immunologic system, nutrition adsorption, and metabolism [1]. Methanogenic archaea are among the anaerobic microorganisms present in the human microbiota [2]. These archaeal species produce methane (CH_4_) by metabolizing hydrogen (H_2_) and carbon dioxide (CO_2_) gases, acetate, formate, and methanol [3, 4].

In humans, the predominant methanogenic archaea are* Methanobrevibacter smithii*, which can comprise up to 10% of all of the anaerobic organisms in the intestinal microbiota [4, 5]. To detect methanogenic archaea in humans, stool samples are cultured [5], examined using molecular biology techniques such as real-time PCR [6, 7] or indirectly by breath methane excretion assessed by gas chromatography [8].

Some studies have associated breath methane excretion with colorectal cancer [9], irritable bowel syndrome [10], diverticulosis [11], and chronic constipation with retentive fecal incontinence [12, 13]. However, the exact role of* M. smithii* in the development or outcome of these illnesses has not yet been established [14]. In the pediatric population, the presence of* M. smithii* in gut microbiota has not been carefully examined. There is little information about CH_4_ production, which is found almost exclusively in children with fecal retentive incontinence secondary to chronic constipation [12, 13, 15].

A previous study [16] reported that a large proportion of children living in a slum near a sanitary landfill were breath CH_4_ producers. In this slum, the environmental concentration of CH_4_ was higher than in locations away from this landfill. The proportion of methane-producing children in this slum was higher than that found in areas with good environmental conditions and was not associated with chronic constipation [16].

Based on these results, the purpose of this study was to evaluate the relationship between living near a sanitary landfill, the socioeconomic and environmental conditions, and the presence of* M. smithii* in the microbiota of children. We evaluated the socioeconomic and environmental conditions, the breath CH_4_ excretion, and the concentration of* M. smithii* in the intestinal microbiota of children living in a slum near a sanitary landfill, in a different slum away from the landfill and students from a high socioeconomic level school.

## 2. Material and Methods

### 2.1. Study Design

This was a community-based, cross-sectional study of children aged 6 to 11 years living in three different socioenvironmental conditions. The study included 111 children living in a slum approximately 50 meters distant from Osasco's sanitary landfill, São Paulo, Brazil, representing approximately 9% of the children in this slum within the age range of the study. The control groups were composed of 35 children from a slum approximately 7.5 kilometers distant from the landfill, representing approximately 11% of the children in this slum within the age range of this study and 32 children from a high socioeconomic level school in the same city, which corresponds to approximately 5% of total students in the age range of this study.

The inclusion criteria considered were age between 6 and 11 years, absence of diarrhea for at least 30 days, nonuse of antibiotics during the 15 days prior to the breath test, and absence of clinical evidence that would characterize serious illnesses such as cardiopathy, nephropathy, type 1 diabetes, or neuropathy. The parents or guardians of children enrolled in the study signed a term of free and informed consent.

### 2.2. Socioeconomic Questionnaire

The socioeconomic characteristics were evaluated through interviews with the parents or guardians using a socioeconomic questionnaire. The variables analyzed were family income, economic class, mother's schooling, demand for health services (public and private), family density, living conditions, and basic sanitation. The division of the families into social classes was performed using the Brazil Economic Classification Criteria.

### 2.3. Breath CH_4_ Dosage

Breath samples were collected from patients after an overnight fast. Moreover, a mouthwash followed by teeth brushing was performed before breath collection. End-expiratory breath samples were collected for testing using a GaSampler system (QuinTron Instrument, Milwaukee, Wisconsin, USA). This apparatus consists of a mouthpiece attached to two bags linked by a T-valve. The first 250 mL of expired air (dead space) enters into a polyvinyl bag, and the valve then automatically shunts, directing the subsequent expired air into the other gas-impermeable bag. The alveolar air sample thus obtained was transferred to a 20 mL plastic syringe with a stopcock. The samples were analyzed immediately with a gas chromatograph MicroLyzer model 12i (QuinTron Instrument, Wisconsin, USA), and the results were expressed in parts per million (ppm). The chromatograph was calibrated using a standard gas mixture containing 92 ppm of hydrogen and 54 ppm of methane (White Martins, Sao Paulo, Brazil).

A child was considered a methane producer if his or her breath methane concentration was greater than or equal to 3 ppm in relation to the methane in the environment [8, 13, 17]. Therefore, air samples were collected in the environment where the breath tests were performed for all three groups.

### 2.4. Stool Collection and DNA Extraction

The stool collection was performed by each child's parents using a clean container and following established guidelines, with the objective of securing bacterial DNA of sufficient quality and quantity. Approximately 1 g of each stool sample was transferred to a microtube containing ASL buffer from a DNA extraction QIAamp Mini Stool Kit (Qiagen, Hilden, Germany) and then frozen at −20°C until the DNA was to be extracted.

The bacterial genomic DNA was extracted according to the protocol recommended by the extraction kit manufacturer (Qiagen, Hilden, Germany). The purified DNA was diluted to a final volume of 200 *μ*L. The DNA concentration was quantified using a NanoDrop 1000 spectrophotometer (Thermo Scientific, Waltham, MA, USA). All of the DNA samples were diluted to a final concentration of 20 ng/*μ*L and stored at −20°C.

### 2.5. Real-Time PCR

In the real-time PCR reactions, a fragment of 151 base pairs (bp) of the* M. smithii*-specific gene* nifH *was used as the target [18]. All of the reactions were performed in duplicate with a final volume of 10 *μ*L containing 5 *μ*L of de Rotor-gene SYBR Green PCR Master Mix (Qiagen, Hilden, Germany), 0.2 *μ*L each of the primers Mnif 202F and Mnif 353R (10 pmol/*μ*L), 0.5 *μ*L of the DNA sample, and 4.1 *μ*L of DEPC-treated water (Qiagen, Hilden, Germany). The thermocycling was performed using a Rotor-gene Q thermocycler (Qiagen, Hilden, Germany) with the following conditions: 95°C for 5 minutes followed by 40 cycles of 95°C for 10 s and 60°C for 15 s, a dissociation cycle for the melting curve of 95°C for 1 minute, and a melting curve program of 70–95°C with a gradual temperature increase of 1°C/s. As a negative control, a reaction containing all of the reagents except the DNA sample was included and its specificity was confirmed by sequencing and alignment using the BLAST system.

The standard curve for all of the analyses was created by amplifying a TopoTA plasmid (Invitrogen) carrying a fragment of the reference gene previously amplified by conventional PCR. With the molecular mass of the plasmid and insert known, it is possible to calculate the copy number as follows: mass in Daltons (g/mol)= (size of double-stranded [ds] product in base pairs [bp]) (330 Da × 2 nucleotides [nt]/bp) [19]. Hence, the g/mol value divided by Avogadro's number equals the g/molecule value, which equals the copy number [19]. Knowing the copy number and concentration of plasmid DNA, the precise number of molecules added to subsequent real-time PCR runs can be calculated, thus providing a standard for specific copy number of genes quantification. The real-time PCR results were expressed as colony forming units/g of feces (CFU/g of feces), once* M. smithii* possesses 1 copy of the* nifH* gene per cell [20].

### 2.6. Statistical Analyses

For the statistical analysis of the numerical variables, either an ANOVA or the Kruskal-Wallis test complemented by the Dunn or Mann-Whitney test, when appropriate, was used. The frequencies and proportions were compared using Pearson's chi-square test (*χ* ^2^) and its partition or Fischer's exact test. The correlations were evaluated using the Spearman coefficient. The alpha error was established as 5%.

## 3. Results

The socioeconomic and environmental data of the studied groups are shown in Table 1. The median age of the participating children was 8.0 years in the slum near the landfill, 8.4 years in the slum away from the landfill, and 8.1 in the high socioeconomic group, with no statistical differences between the groups (*P* = 0.317). A statistically significant difference was found among the 3 groups with respect to the type of housing and electrical energy supply. The frequency of a brick house and regularized electricity was higher in the slum near the landfill than in the slum away from the landfill. The presence of a sewage network, water supply, paved street, and earthen backyard as well as the income per capita and distribution of social classes was similar between the two slum groups, whereas the control high socioeconomic group was significantly better off to all of the evaluated aspects than either of the 2 slum groups.

The environmental air samples collected in the slum near the sanitary landfill, the slum away from the landfill, and the high socioeconomic school environment contained 9 ppm, 1 ppm, and 0 ppm of CH_4_, respectively. The cut-off point from which a child was considered as CH_4_ producers in the slum near the landfill is 12 ppm of breath CH_4_, in the slum away from landfill more than 4 ppm and above 3 ppm in the high socioeconomic group.  Table 2 shows the prevalence of methane producer's children and the median concentration of breath CH_4_ in all 3 groups. The median concentration of breath methane was higher in children living near the landfill than in the other two groups.

Using real-time PCR,* M. smithii* was detected in all of the stool specimens from the three groups.  Table 3 presents the quantitative results of* M. smithii* concentrations in the fecal microbiota of CH_4_-producers and non-CH_4_ producers in the 3 studied groups. The total concentration of* M. smithii* in the feces of the children living in the slum near the sanitary landfill was higher than that found in the other two groups.

The correlation coefficient between the breath CH_4_ concentration and the fecal concentration of* M. smithii* was +0.556 (*P* < 0.001) for the children from the slum near the sanitary landfill, +0.754 (*P* < 0.001) for the children living in the slum away from the sanitary landfill, and +0.464 (*P* = 0.007) for the children in the high socioeconomic group.

## 4. Discussion

The real-time PCR results demonstrated the presence of* M. smithii* in the stools of all of the children who participated in this study. As we expected, the group of children living in the slum near the sanitary landfill had a higher concentration of* M. smithii* in their stools and greater prevalence of breath CH_4_ producers that agrees with our previous study [16]. This result is also in agreement with methane concentration in the environment air; however the fecal* M. smithii* concentrations of the children living in a slum away from the landfill were similar to those observed in children living in better socioeconomic conditions.

*M. smithii* is difficult to grow* in vitro*; therefore, molecular methods that test for the presence of 16S rRNA genes and other* M. smithii-*specific genes, such as* nifH*, have become popular [6, 7, 21]. The successful use of the* nifH* gene in the detection of* M. smithii* in contaminated water has demonstrated the high specificity and sensibility of such an approach, making it a good target gene for identification and quantification [18, 21]. With the use of real-time PCR, another study quantified the presence of* M. smithii* in the microbiota of obese, normal, and anorexic adult patients, demonstrating the presence of* M. smithii* in approximately 80% of the samples with average concentrations varying from 9.78 × 10^7^ to 1.68 × 10^8^ copies/g of feces [22]. Dridi et al. [6] tested for the presence of* M. smithii* in 700 stool samples from children and adults using real-time PCR and found that 95.7% of the samples were positive, with concentrations varying between 1.09 × 10^1^ and 1.45 × 10^11^ copies/g of feces. Stewart et al. [7] found concentrations of* M. smithii* ranging from 7.45 × 10^5^ to 4.91 × 10^7^ CFU/g of feces from 12 adults and 40 children's samples using real-time PCR assays. Our study found concentrations of* M. smithii* ranging from 10^4^ to 10^8^ CFU/g of stool, which is consistent with the data in the studies described above. On the other hand, Weaver et al. [11] found concentrations of* M. smithii* ranging from 1.0 × 10^7^ to 3.0 × 10^10^ CFU/g of feces from 130 adults before sigmoidoscopy, including individuals with normal colon, diverticulosis, inflammatory bowel disease, colon polyps, and colon cancer using culture methods.

*M. smithii* was evaluated on fecal microbiota through molecular methods only in France [6, 22] and New Zealand [7]; this is the first study in Brazil evaluating the presence of* M. smithii* on pediatric populations with different socioeconomic conditions. The studies in Brazil have used only the dosage of breath CH_4_ as an indirect marker for the presence of methanogenic archaea in children with severe chronic constipation in a specialized outpatient clinic of pediatric gastroenterology [12, 13] and children living in different environmental conditions [16].

The concentration of breath CH_4_ is used to classify a population into CH_4_ producers and CH_4_ nonproducers [8]. Considering only this criterion, the prevalence of children considered to be CH_4_ producers reported in the literature varies between 6% and 40% [23, 24], whereas among adults, the reported prevalence of producers is higher, varying from 33% to 70% [8, 17, 24]. In the present study, the prevalence of CH_4_ producers was 53.1% among the children residing in the slum near the sanitary landfill; this percentage was higher than that found in the children living in the slum away from the landfill and children in the higher socioeconomic group (31.4% and 21.9%, resp.).

The prevalence of CH_4_-producing children encountered in the two control groups who live away from the landfill was similar to the range of 14.3 to 18.2% reported in a study in Israel that included children aged 7 to 14 [23]. In another study conducted in a rural population in Nigeria, the prevalence of breath CH_4_ producers between the ages of 2 and 6 was 40% [24]. Taken together, the findings of these studies suggest that there are differences in the proportions of breath CH_4_ producers in groups from different socioeconomic classes who live in different environmental conditions.

The present study showed the presence of* M. smithii* in all fecal samples analyzed, even in those children without breath CH_4_ excretion. It should be emphasized that it was not possible to establish a cut-off point between the microbiota concentration of* M. smithii* and the minimum breath CH_4_ concentration detected. The correlation between breath CH_4_ excretion and the microbiota concentration of* M. smithii* varied among the studied groups. For the children residing in the slum away from the sanitary landfill, a stronger correlation was found between breath CH_4_ excretion and the* M. smithii* being better than that observed for the children in the high socioeconomic level group. Approximately 20% of all of the CH_4_ produced in the gastrointestinal tract is expelled through the lungs [8]; this can explain the differences found in the correlation between the breath CH_4_ and the concentration of* M. smithii* in the microbiota of the children.

The children living near the sanitary landfill show higher concentrations of* M. smithii* in microbiota and breath CH_4_ compared with the other groups. Among all the children considered breath CH_4_ producers the concentration of* M. smithii* in the microbiota was similar in the three groups. The children living near the sanitary landfill present higher counts of* M. smithii* in the microbiota, even when they do not produce breath CH_4_. Despite the socioeconomic differences, the children living in the slum away from the landfill and those from the high socioeconomic group had similar fecal concentrations of* M. smithii* between the non-CH_4_ producers. The children living near the sanitary landfill were classified into CH_4_ with breath excretion above 12 ppm of CH_4_ because of the high concentration of CH_4_ in the environment, while in the other groups the cut-off point was 4 ppm and 3 ppm, which can explain the higher counts of* M. smithii* among the nonproducers CH_4_ from the slum near the sanitary landfill.

Residing close to sanitary landfills can increase exposure to microorganisms and toxic gases emitted by these types of installations [25]. In Finland, studies have been conducted on two different sanitary landfills, evaluating the concentration of microorganisms and gases in the surrounding air [26]. The researchers found that the concentrations of bacteria and viable fungi dispersed in the air into those sanitary landfills were approximately 5 to 20 times higher than those found in the exterior environment [26].

The development of methanogens is not directly related to the introduction of particular foods and the main factors that influence the occurrence of methanogenics archaea are the environment factors [27]. By analyzing the socioeconomic and environment conditions found among the groups, we can deduce that living near a sanitary landfill was a major factor contributing to the differences observed in the microbiota of the children. The children living in the slum away from the sanitary landfill and those who live near the sanitary landfill present the same socioeconomics conditions, including income per capita and basic sanitation, while the high socioeconomic group presents better conditions for both characteristics.

Increased concentration of breath CH_4_ and* M. smithii* in the intestinal microbiota has been associated with diverticulosis [11], constipation-predominant irritable bowel syndrome [10], and chronic constipation with retentive fecal incontinence in children [13]. Pimentel et al. have also shown that CH_4_ slows intestinal transit and augments small intestinal contractile activity and these contractions are isolated, segmental, and nonpropagating [28].* M. smithii* can interact with other bacteria of the gut microbiota, enhance the activities and growth of polysaccharide consumers like* Bacteroidetes* and* Firmicutes* by removing H_2_, and promote caloric intake [29].

The role* M. smithii* might have in pathological conditions is still unclear, but through syntrophic interactions methanogens might support the growth of fermenting bacteria, which themselves could be either true pathogens or at least opportunistic pathogens which influence our health in other indirect ways [30, 31]. The children living near the sanitary landfill possess significant increase of* M. smithii* in the microbiota, which can cause microbiota's alterations that might influence their health in the future; their pattern of microbiota may help to clarify which bacteria are more sensible to variations of* M. smithii* concentrations through further investigations.

## 5. Conclusion

The present study is the first report showing the distribution of* M. smithii* in children living in different socioeconomics conditions in Brazil and worldwide. To live near a sanitary landfill is associated with higher concentrations of* M. smithii* in intestinal microbiota, comparing with those who live away from the sanitary landfill, regardless of their socioeconomics conditions. The effects of these changes cannot be seen in the health of the children; however investigating the alterations on microbiota of peoples living in those conditions could help to understand the relations between* M. smithii* and other microorganisms.

## Figures and Tables

**Table 1:** Socioeconomic and environmental conditions of children living in the three distinct areas.

	Slum near landfill group		Slum away from the landfill group		High socioeconomic group		*P* ^1^
	*n*	%	*n*	%	*n*	%	
Brick house	69^a^	63.4%	11^b^	31.4%	32^c^	100.0%	<0.001
Sewage network	10^a^	9.2%	4^a^	11.4%	32^b^	100.0%	<0.001
Piped water	60^a^	55.5%	23^a^	65.7%	32^b^	100.0%	<0.001
Regularized electricity	70^a^	64.9%	1^b^	2.9%	32^c^	100.0%	<0.001
Paved street	14^a^	13.0%	0^a^	0.0%	32^b^	100.0%	<0.001
Backyard of land	39^a^	36.1%	15^a^	42.9%	0^b^	0.0%	<0.001
Pets	52^a^	48.1%	8^b^	22.9%	18^a^	46.1%	0.019
Income per capita >1/2 MW	22^a^	20.4%	2^a^	5.7%	32^b^	100.0%	<0.001
Social class							
A	0^a^	0.0%	0^a^	0.0%	9^b^	26.5%	
B	4^a^	3.7%	0^a^	0.0%	20^b^	58.8%	
C	67^a^	62.0%	17^a^	48.6%	5^b^	14.7%	<0.001
D	31^a^	28.7%	18^a^	51.4%	0^b^	0.0%	
E	6^a^	5.6%	0^a^	0.0%	0^b^	0.0%	

^1^Chi-squared test: different letters on the line represent statistically significant differences (*P* < 0.05); MW = minimum wage.

**Table 2:** Prevalence of breath CH_4_ producers and children breath CH_4_ concentration (ppm) in the three distinct groups.

	Slum near the landfill	Slum away from the landfill	High socioeconomic group	*P*
Breath CH_4 _producer prevalence	53.1% (59/111)^a^	31.4% (11/35)^b^	21.9% (7/32)^b^	0.001^2^
CH_4_ in breath CH_4_ producers	24 (18.0–35.0)^a^	17.0 (10.0–31.0)^b^	17 (11.0–19.0)^b^	0.007^1^
Total breath CH_4_	14 (0.0–25)^a^	2.0 (0.0–9.5)^b^	0.0 (0.0–2.5)^b^	<0.001^1^

^1^Mann-Whitney test: the median and percentiles 25 and 75; ^2^Chi-squared test; different letters on the lines represent statistically significant differences (*P* < 0.05).

**Table 3:** Comparison of *M. smithii* concentrations in CFU/g of feces, in accordance with breath CH_4_ production in the three distinct groups.

CH_4_ Producer	Slum near the landfill		Slum away from landfill		High socioeconomic group		*P*
	*n*	Concentration	*n*	Concentration	*n*	Concentration	
Yes	59	4.26 × 10^7^	11	1.42 × 10^7^	7	1.26 × 10^7^	0.094^1^
		(1.41 × 10^7^–1.46 × 10^8^)		(7.97 × 10^6^–7.39 × 10^8^)		(5.32 × 10^5^–3.94 × 10^7^)	
No	52	1.64 × 10^7a^	24	5.63 × 10^5b^	25	2.06 × 10^5b^	<0.001^1^
		(2.83 × 10^6^–5.42 × 10^7^)		(6.13 × 10^4^–2.97 × 10^6^)		(3.11 × 10^4^–2.59 × 10^6^)	

Total	111	3.16 × 10^7a^	35	2.05 × 10^6b^	32	3.93 × 10^5b^	<0.001^1^
		(6.36 × 10^6^–8.63 × 10^7^)		(1.55 × 10^5^–1.66 × 10^7^)		(5.05 × 10^4^–5.54 × 10^6^)	

^1^One-way ANOVA complemented by Dunn's Test, median and percentiles 25 and 75; Different letters in the same line represent statistically significant differences: *P* < 0.05.
